# Contemporary Evaluation and Management of the Diabetic Foot

**DOI:** 10.6064/2012/435487

**Published:** 2012-10-09

**Authors:** Bauer E. Sumpio

**Affiliations:** Department of Vascular Surgery, Yale University School of Medicine, New Haven, CT 06510, USA

## Abstract

Foot problems in patients with diabetes remain a major public health issue and are the commonest reason for hospitalization of patients with diabetes with prevalence as high as 25%. Ulcers are breaks in the dermal barrier with subsequent erosion of underlying subcutaneous tissue that may extend to muscle and bone, and superimposed infection is a frequent and costly complication. The pathophysiology of diabetic foot disease is multifactorial and includes neuropathy, infection, ischemia, and abnormal foot structure and biomechanics. Early recognition of the etiology of these foot lesions is essential for good functional outcome. Managing the diabetic foot is a complex clinical problem requiring a multidisciplinary collaboration of health care workers to achieve limb salvage. Adequate off-loading, frequent debridement, moist wound care, treatment of infection, and revascularization of ischemic limbs are the mainstays of therapy. Even when properly managed, some of the foot ulcers do not heal and are arrested in a state of chronic inflammation. These wounds can frequently benefit from various adjuvants, such as aggressive debridement, growth factors, bioactive skin equivalents, and negative pressure wound therapy. While these, increasingly expensive, therapies have shown promising results in clinical trials, the results have yet to be translated into widespread clinical practice leaving a huge scope for further research in this field.

## 1. Introduction

 Approximately, 26 million people, comprising 8.3% of the US population are estimated to have diabetes [1]. In 2010 alone, there were 2 million new cases of diabetes diagnosed. This becomes even more significant with increasing age as the prevalence of diabetes increases to 27% in the population over age 65 [1]. The American Diabetes Association consensus group identified increased risk in patients with diabetes for >10 years, which are males, having poor glucose control or having cardiovascular, retinal, or renal complications [2].Of the US diabetic population, it is estimated that 15% will develop manifestations of diabetic foot disease in their lifetime [3]. Foot ulcers are common in patients with diabetes mellitus with a prevalence as high as 25% and an annual incidence of 2%-3% [3]. Although cancer and trauma can result in amputations, chronic diabetic foot ulcers lead to more than 80% of nontraumatic amputations and account for 46% of the 162,000 hospital admissions for foot ulcers annually [3]. This prevalence of foot disease in the diabetic population results in significant clinical and economic impact. Data from the National Hospital Discharge Survey demonstrates that approximately 51% of nontraumatic lower extremity amputations are performed on diabetics [4] with an age adjusted amputation rate that is 15%–40% higher than nondiabetics [4]. It is estimated that 15% of Americans with diabetes will develop manifestations of diabetic foot disease in their lifetime [3, 5].

 Minor trauma, often footwear related and exacerbated by prolonged pressure and local infection, is a frequent inciting event. Infection is a frequent (40%–80%) and costly complication of these ulcers and presents a major cause of morbidity and mortality. Diabetic foot ulcers and their sequel amputations, besides being the most serious and expensive complications of diabetes, are a major cause of disability, morbidity, and mortality for these patients [6]. A detailed knowledge of the clinical picture, pathogenesis, relevant diagnostic tests, and treatment modalities is essential in planning the optimal treatment strategy for diabetic ulcers. An incorrect or delayed initial diagnosis may increase the risk of serious complications, including permanent disability and amputations.

## 2. Biomechanics of Walking and Ulcer Formation

**Figure 1:**
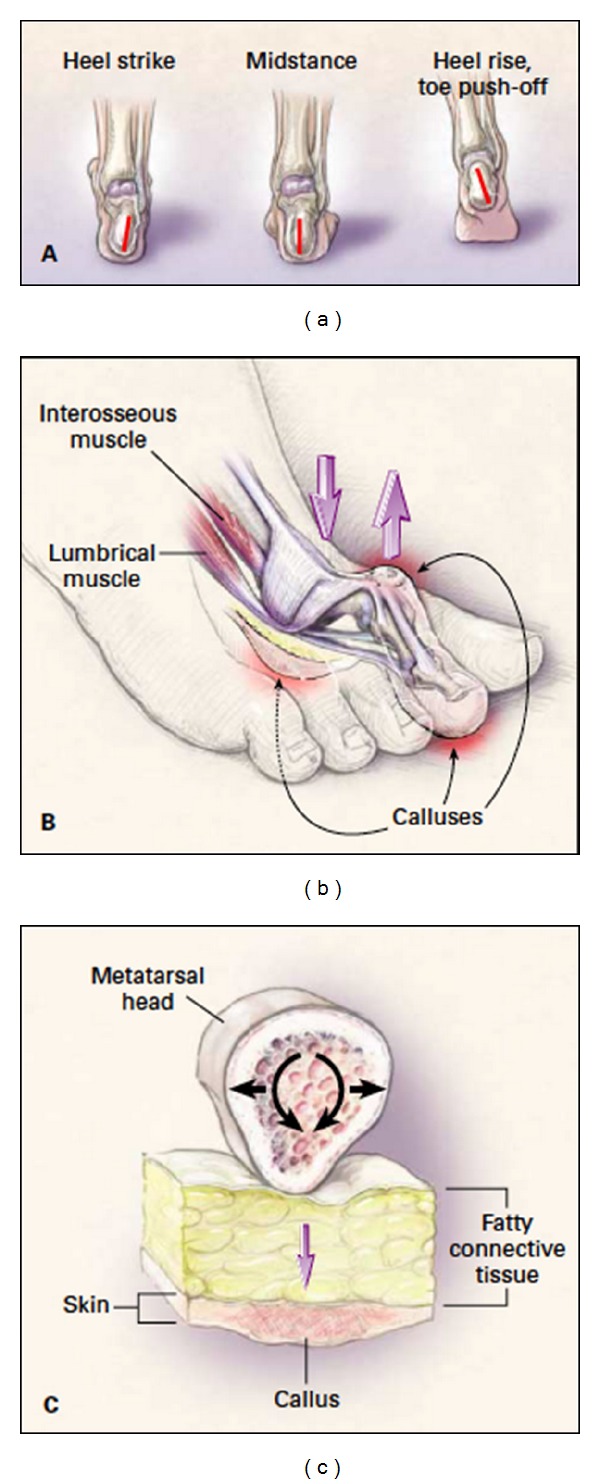
Biomechanics of foot ulcers. (a) The biomechanics of gait. The first action is heel strike, when the lateral calcaneus makes contact with the ground and the muscles, tendons, and ligaments relax, providing for optimal energy absorption. The second is midstance, when the foot is flat and is able to adapt to uneven terrain, maintain equilibrium, and absorb the shock of touchdown. The third is heel rise followed by toe push-off, when the calcaneus lifts off the ground, the foot pronates, the muscles, tendons, and ligaments tighten, and the foot regains its arch. (b) The forces on the foot. (c) Callus formation. Adapted from NEJM [9] with the permission of the publisher.

**Figure 2:**
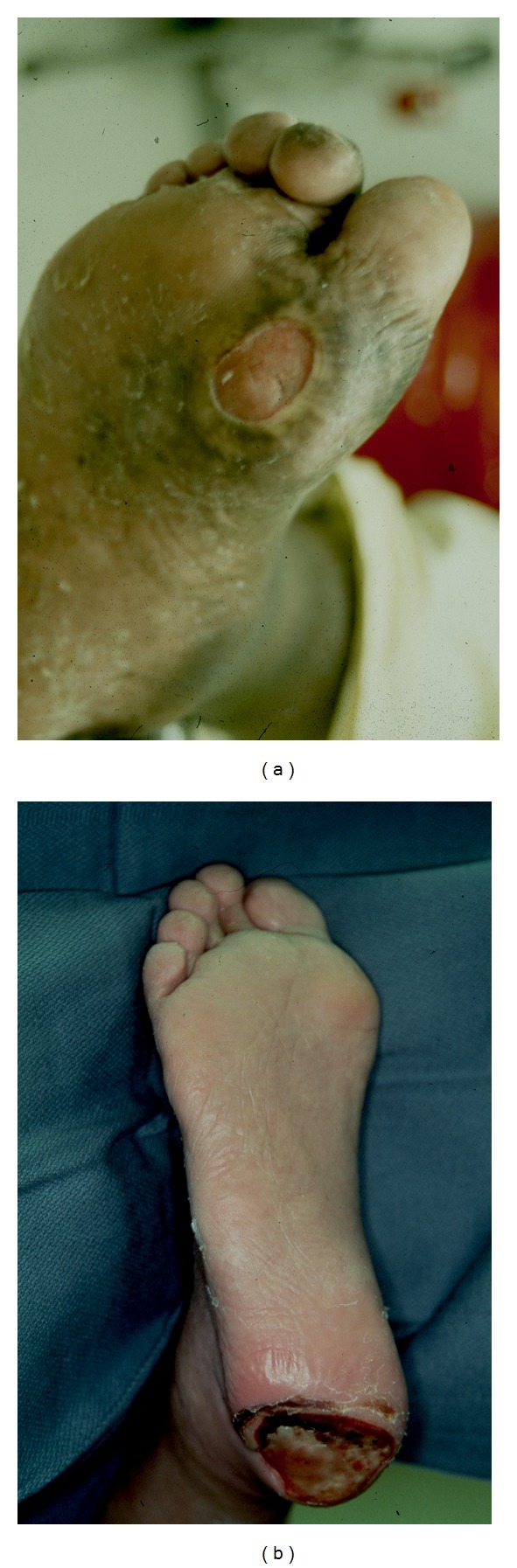
(a) Mal perforans ulcer over the first metatarsal head. (b) Mal perforans ulcer at the heal.

The foot is a complicated biologic structure containing 26 bones, numerous joints, and a network of ligaments, muscles, and blood vessels. An appreciation of the biomechanics required for walking is essential in understanding the etiology of foot ulcers (Figure 1). Gait is a complex set of events that requires triplanar foot motion and control of multiple axes for complete bipedal ambulation [7–9]. Various external and internal forces affect foot function. The combination of body weight pushing down and the ground reactive force pushing up creates friction and compressive forces. Shear results from the bones of the foot sliding parallel to their plane of contact during pronation and supination. Foot deformities or ill-fitting footwear enhance pressure points because they focus the forces on a smaller area. When the foot flattens too much or overpronates, the ankle and heel do not align during midstance and some bones are forced to support more weight. The foot strains under the body's weight, causing the muscles to pull harder on these areas, making it more difficult for tendons and ligaments to hold bones and joints in proper alignment. Over time, swelling and pain on the bottom of the foot or near the heel may occur. Bunions can form at the great toe joint, and hammertoe deformities can form at the lesser toes. Abnormal foot biomechanics resulting from limited joint mobility and foot deformities magnify shearing forces, resulting in increased plantar pressure on the foot during ambulation. This can represent critical causes for tissue breakdown. For instance, ischemic ulcers often develop on the dorsum of the foot, over the first (Figure 2(a)) and fifth metatarsal heads. A heel ulcer can develop from constant pressure applied while the heel is in a dependent position or during prolonged immobilization and bed rest (Figure 2(b)). Although simple cutaneous breakdown is not infrequent because of shearing forces or direct trauma, healing is the rule unless the wound repair mechanisms are suboptimal due to impairment of perfusion, infection, or repeated, continuous traumatic insults. Lack of sensation allows the damage to cascade to ulceration. Lack of perfusion decreases tissue resilience and leads to rapid death of tissue and impedes wound healing for tissue repair. Broadly speaking, therefore, the progression to foot ulceration can be attributed to impaired arterial supply, neuropathy, musculoskeletal deformities, infection, or a combination of these factors.

## 3. Etiology of Diabetic Foot Ulceration

### 3.1. Arterial Disease

 The incidence of lower extremity ulcers caused by peripheral arterial disease is increasing in Western nations [10]. The general “aging” of the population and better diagnostic techniques may provide possible explanations for this observation. Risk factors for the development of atherosclerotic lesions causing leg ischemia include diabetes mellitus, smoking, hyperlipidemia, hypertension, obesity, and age [2]. Lack of perfusion decreases tissue resilience, leads to rapid death of tissue, and impedes wound healing. Wound healing and tissue regeneration depend on an adequate blood supply to the region. Ischemia due to vascular disease impedes healing by reducing the supply of oxygen, nutrients, and soluble mediators that are involved in the repair process [11]. Purely ischemic diabetic foot ulcers are uncommon, representing only 10% to 15% of ulcers in patients with diabetes. More commonly, ulcers have a mixed ischemic and neuropathic origin, representing 33% of diabetic foot ulcers [12]. Once an ulcer is formed, the blood supply necessary to allow healing of the wound is greater than that needed to maintain intact skin. This leads to chronic ulcer development unless the blood supply is improved.

 Peripheral arterial disease is estimated to be 2 to 4 times more common in persons with diabetes than in others [13]. Compared to general population, diabetics are affected by atherosclerosis at a younger age, the atherosclerosis tends to progress at a much faster rate and results in higher rates of amputation [14, 15]. Its hallmark is the involvement of the tibioperoneal vessels with relative sparing of the pedal vessels. Characteristically, diabetic occlusive lesions spare the arteries above the knee but involve the infrapopliteal arteries with calcific single or multiple level disease. In more than 90% of patients, one or more of the large vessels at the ankle and in the foot are spared. In most cases, the peroneal artery in the calf remains patent and is the last of the three crural arteries to occlude prior to which it continues to provide pedal circulation via its terminal branches. Consequently, bypass to a single tibial or peroneal artery or a pedal bypass has the potential to provide good blood flow to the foot. Occlusive lesions affecting the foot and precluding revascularization are not common in diabetic patients [5]. The presence of multisegment disease, the frequency of calcification of both the atherosclerotic lesions and the wall of the arteries involved, and the presence of renal insufficiency are additional challenges often encountered. Incompressibility of extremity arteries makes ankle brachial indices misleading in the assessment of the severity of the vascular disease. 

### 3.2. Neuropathy

 Neuropathy is the most common etiology underlying foot ulceration and frequently involves the somatic and autonomic fibers. Although there are many causes of peripheral neuropathy, diabetes mellitus is by far the most common. Neuropathy, usually distal sensorimotor polyneuropathy, is present in about 42% of diabetic patients after 20 years [11]. The peripheral neuropathy is thought to result from abnormalities in metabolic pathways, of which there are several hypotheses including deficiencies in sorbitol metabolism via the polyol pathway [12, 16]. 

 Type-A sensory fibers are responsible for light touch, vibration, pressure, proprioception, and motor innervations to the intrinsic muscles of the foot. Type-C sensory fibers detect painful stimuli, noxious stimuli, and temperature. When these fibers are affected, protective sensation is lost which manifests as a distal, symmetric loss of sensation described as a “glove and stocking” distribution and proves to be the primary factor predisposing patients to ulcers and infection [17]. Patients are unable to detect increased loads, repeated trauma, or pain from shearing forces. Injuries such as fractures, ulceration, and foot deformities therefore go unrecognized. Repeat stress to high-pressure areas or bone prominences, which would be interpreted as pain in the nonneuropathic patient, also goes unrecognized. Sensory dysfunction results in increased shearing forces and repeated trauma to the foot [18, 19]. 

 Neurotrophic ulcers typically form on the plantar aspect of the foot at areas of excessive focal pressures. This is mostcommonly encountered over the bony prominences of the metatarsal heads and the forefoot region due to the requirements of midstance and heel off during the gait cycle. Several investigators have demonstrated that there is an increase in both static and dynamic foot pressures in a neuropathic foot [18, 20, 21]. 

 Three mechanisms frequently play a part in the development of mechanically induced neuropathic ulcerations. The first mechanism is usually the result of a quick traumatic event, like stepping on a sharp object such as a nail or a piece of broken glass, which results in piercing of the skin. The second mechanism is application of chronic low grade pressure as would be seen with wearing an ill-fitting shoe. This creates focal areas of tissue ischemia over a bony prominence such as a bunion or hammer toe. If the pressure is maintained for a significant period of time, this leads to necrosis and ulceration. The third mechanism involves a force of repetitive, moderate pressure. This accounts for the majority of diabetic plantar ulcers. Repetitive pressure >10 kg per cm acting on the foot during gait will contribute to the formation of this type of ulcer. In the absence of neuropathy, the repetitive pressure beneath prominent areas produces pain that prompts a sensate person to take measures to alleviate the discomfort. Such measures include limping or modification of gait to place weight on a different part of the foot, changing to more comfortable shoes, applying pads, or seeking medical treatment. 

 The loss of protective sensation in the neuropathic patient lets foot wounds go undetected resulting in areas of inflammation and enzymatic autolysis culminating in tissue breakdown and ulceration. The location of this type of ulcer is predictable. Areas of increased pressure are commonly identified by areas of plantar callus formation, which conversely are the areas prone to ulceration in a neuropathic patient. Patients have inadequate protective sensation during all phases of gait; therefore, high loads are undetected due to loss of pain threshold, which results in prolonged and increased forces [22]. These problems manifest as abnormal pressure points, increased shearing, and greater friction to the foot. Because this goes unrecognized in the insensate foot, gait patterns remain unchanged, and the stresses eventually cause tissue breakdown and ulceration. To date, high pressures alone have not been shown to cause foot ulceration. Rheumatoid patients with high plantar foot pressures but no sensitivity deficit have almost no evidence of foot ulceration [23]. Loss of protective sensation secondary to neuropathycan rapidly lead to ulceration at these high pressure zones if patient education and preventive measures are not taken.

 Motor neuropathy is associated with demyelinization and motor endplate damage. The distal motor nerves are the most commonly affected, resulting in atrophy of the small intrinsic muscles of the foot. Wasting of lumbrical and interosseous muscles of the foot results in collapse of the arch and loss of stability of the metatarsophalangeal joints during midstance of the gait. Overcompensation by extrinsic muscles can lead to musculoskeletal deformities [24]. Autonomic involvement causes an interruption of normal sweating at the epidermal level and causes arteriovenous shunting at the subcutaneous and dermal level. Hypohidrosis leads to a noncompliant epidermis that increases the risk of cracking and fissuring. Arteriovenous shunting diminishes the delivery of nutrients and oxygen to the tissues making them susceptible to breakdown [25].

**Figure 3:**
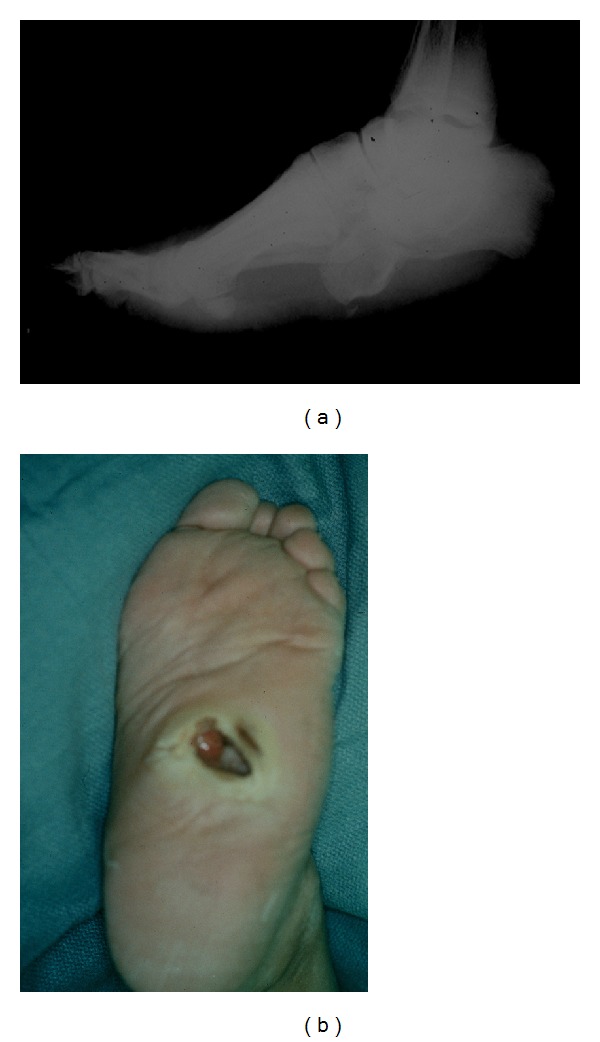
Rocker bottom foot deformity secondary to Charcot disease. (a) X-ray image lack of ankle joint dorsiflexion resulting in an increased load to the forefoot. This in turn leads to collapse of the tarsometatarsal joint, which is the focus point between the forefoot and hindfoot. The arch has collapsed and appears to be almost inverted. (b) Charcot foot with rocker bottom has increased propensity to pressure ulcers formation and is often seen with the involvement of Lisfranc joint.

 Diabetic patients are especially prone to development of a neuroosteoarthropathy also known as Charcot foot [20]. This condition is thought to involve autonomic nerve dysfunction resulting in abnormal perfusion to foot bones leading totheir fragmentation and collapse. The resulting “rocker bottom foot” is prone to tissue breakdown and ulceration (Figure 3) [5, 22]. 

### 3.3. Musculoskeletal Deformities

**Figure 4:**
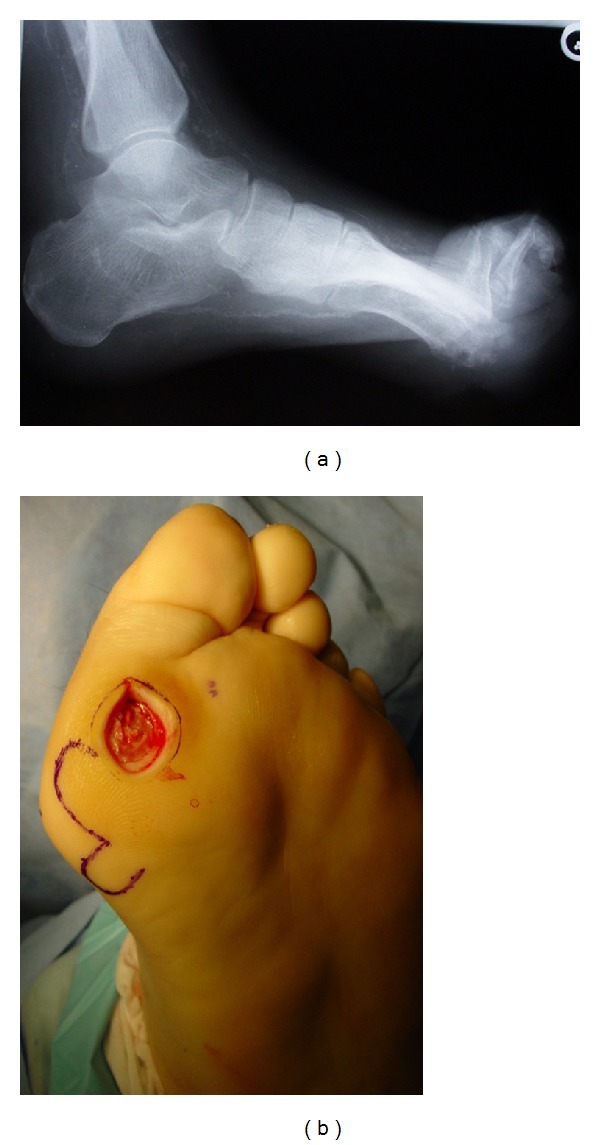
Abnormal biomechanics contributing to pressure ulcer at first metatarsophalngeal joint. (a) X-ray image showing foot deformity. (b) Photograph of foot illustrating bony abnormalities and ulceration.

 Four foot-related risk factors have been identified in the genesis of pedal ulceration: altered biomechanics, limited joint mobility, bony deformity, and severe nail pathology [2]. Atrophy of the small muscles within the foot results in nonfunctioning intrinsic foot muscles referred to as an “intrinsic minus foot” [8]. The muscles showing early involvement are the flexor digitorum brevis, lumbricals, and interosseous muscles. This group acts to stabilize the proximal phalanx against the metatarsal head preventing dorsiflexion at the metatarsophalangeal joint (MTPJ) during midstance in the gait cycle. With progression of the neuropathy, these muscles atrophy and fail to function properly. This causes the MTPJs to become unstable, allowing the long flexors (flexor digitorum longus and flexor hallucis longus) and extensors (extensor digitorum longus and extensor hallucis longus) to act unchecked on the digits. Dorsal contractures develop at the MTPJs with development of hammer digits (Figure 4). The deformity acts to plantar flex the metatarsals, making the heads more prominent and increasing the plantar pressure created beneath them. It also acts to decrease the amount of toe weight bearing during the gait cycle, which also increases pressure on the metatarsal heads. In essence, a mobile adapter is converted to a rigid lever. Pressure is equal to body weight divided by surface area, thus decreasing surface area below a metatarsal head with concomitant rigid deformities and leading to increased forces or pressure to the sole of the foot. A low pressure but constant insult over an extended period can have the same ulcerogenic effect as high pressure over a shorter period. This is typical of the effect of tight-fitting shoes. If the magnitude of these forces in a given area is large enough, either skin loss or hypertrophy of the stratum corneum (callus) occurs. The presence of callus in patients with neuropathy should raise a red flag because the risk of ulceration in a callused area is increased by two orders of magnitude [9]. 

**Figure 5:**
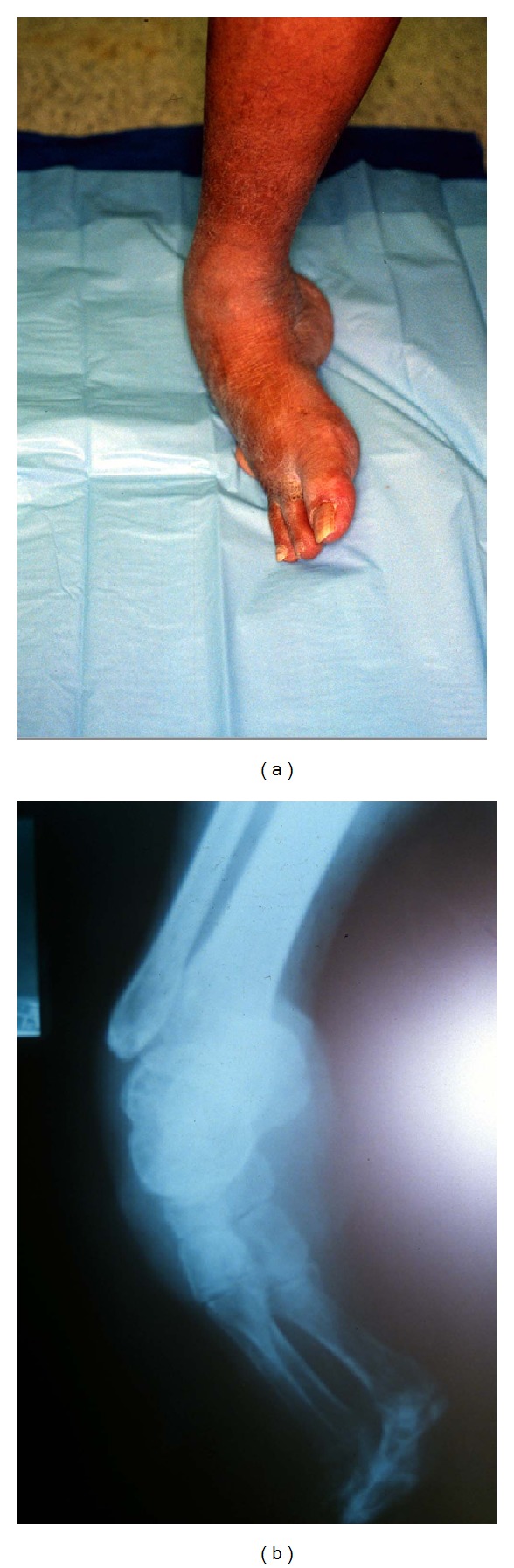
(a) Overpowering of the extrinsic foot muscles leading to an equinus deformity at the ankle and a varus hindfoot. (b) X-ray image showing ankle and foot deformity.

 Overpowering by the extrinsic foot muscles also leads to an equinus deformity at the ankle and a varus hindfoot. A cavovarus foot type can develop, leading to decreased range of motion of the pedal joints, an inability to adapt to terrain, and low tolerance to shock (Figure 5). In patients with “flatfoot” deformities, there is often excessive pronation and a hypermobile first ray that leads to an excessive amount of pressure beneath the second metatarsal. In neuropathic patients with this foot type, callus formation and subsequent ulcerations often develop beneath the second metatarsal head. In contrast, patients with a rigid cavus foot commonly ulcerate beneath the heel, the first metatarsal, and/or the fifth metatarsal. In patients with a “rocker bottom” Charcot deformity, the area beneath the cuboid is an area of increased risk (Figure 3). 

### 3.4. Infection

 Patients with diabetes appear to be more prone to various infections than their nondiabetic counterparts [26]. 40%–80% of diabetic foot ulcers have evidence of infection. Several factors increase the risk of development of diabetic foot infections including diabetic neuropathy, peripheral arterial disease, and immunologic impairment. Diabetic state causes impairment in the functioning of polymorphonuclear leukocytes that can manifest as a decrease in migration, phagocytosis, and decreased intracellular activity. Evidence suggests impaired cellular immune response, as well as abnormalities in complement function [12, 16]. Some of the defects appear to improve with control of hyperglycemia underscoring the need for a tight and consistent control of hyperglycemia.

 Undiagnosed clean neuropathic foot ulcers often convert to acute infections with abscess and/or cellulites [27]. Most infections involve soft tissues of the foot, but about 20% of the patients develop culture-positive osteomyelitis. Presence of peripheral arterial disease, neuropathy, or impaired leukocyte functions may reduce the local inflammatory response and classical signs or symptoms of local infection that makes the diagnosis of infection in a diabetic foot especially challenging. 

 Diabetic foot infections can be classified into those that are nonthreatening and those that are life or limb threatening. Non-limb-threatening diabetic foot infections are often mild infections associated with a superficial ulcer. They often have less than 2 cm of surrounding cellulitis and demonstrate no signs of systemic toxicity. These infections have on average 2.1 organisms [27, 28]. Aerobic gram-positive cocci are the sole pathogens in 42% of these cases, with the most notable organisms being *Staphylococcus aureus*, coagulase-negative *S. aureus*, and *streptococci*. These less severe infections can often be managed with local wound care, rest, elevation, and oral antibiotics on an outpatient basis. A foot infection in a diabetic patient can present with a more severe, life- or limb-threatening picture. In these patients, there is usually a deeper ulceration or an undrained abscess, gangrene, or necrotizing fasciitis. Methicillin-resistant *Staphylococcus aureus* (MRSA) is an increasingly common isolate [27–29]. They tend to have greater than 2 cm of surrounding cellulitis, as well as lymphangitis and edema of the affected limb. These more severe cases generally present with fever, leukocytosis, and hyperglycemia.

 In contrast to nondiabetic individuals, complex foot infections in diabetic patients usually involve multiple organisms with complex biofilm environments [30]. Studies report an average of five to eight different species per specimen [29, 31–33]. These included a combination of gram-positive and -negative, as well as aerobic and anaerobic organisms. The most prevalent organisms identified were *S. aureus*, coagulase-negative *Staphylococcus*, group B *Streptococcus*, *Proteus*, *Escherichia coli*, *Pseudomonas*, and *Bacteroides*. Recently, methicillin-resistant *S. aureus* (MRSA) infection has become more common in diabetic foot ulcers and is associated with previous antibiotic treatment and prolonged time to healing [29, 32–34]. Anaerobic infections with Clostridium are also not uncommon. 

## 4. Assessment of Diabetic Foot Ulcer

 Accurate diagnosis of the underlying cause of lower extremity ulceration is essential for successful treatment. The etiology of most leg ulcers can be ascertained quite accurately by careful, problem-focused history and physical examination [9]. Diagnostic and laboratory studies are occasionally necessary to establish the diagnosis but are more often performed to guide treatment strategy [35].

### 4.1. History

 Arterial insufficiency is suggested by a history of underlying cardiac or cerebrovascular disease, complaints of leg pain when walking, or impotence. Symptoms of arterial insufficiency occur because of inadequate perfusion to the lower extremity relative to its metabolism. Tissue hypoxia and the subsequent increase in concentration of lactic acid produce pain. Patients may complain of pain in the buttocks or calves brought on with activity and relieved with rest (intermittent claudication) or pain in the forefoot aggravated by elevation and relieved by dependency (rest pain). The presence of an extremity ulcer is an easily recognized but late sign of peripheral vascular insufficiency. Patients with lower extremity ulcers resulting from atherosclerotic disease usually have a risk-factor profile that includes: older age, male sex, smoking, diabetes mellitus, hypertension, hypercholesterolemia, and obesity. Patients with leg ulcers and multiple atherosclerotic risk factors often have atherosclerosis in other arterial beds [26]. Up to one-third of patients with diabetes mellitus can have significant atherosclerotic disease, without specific symptoms. Most common complaints are those of neuropathic disease, which include history of numbness, paresthesias, and burning pain in the lower extremities. Patients often report previous episodes of foot ulcers and chronic skin infections.

### 4.2. Physical Examination

 A complete examination can only be performed with the patient supine in an examination gown [36]. The patient's vital signs are recorded and abnormalities noted. The patient's temperature, respiratory rate, heart rate, and blood pressure in both upper extremities should be obtained. Fever may indicate the presence of an infected ulcer, and the presence of tachycardia and tachypnea may support the diagnosis of a septic foot.

 A classic look, listen, and feel examination includes inspection of the skin of the extremities, palpation of all peripheral pulses, measurement of ankle-brachial indices, assessment of extremity temperature, auscultation for bruits, and a thorough neurologic examination [37]. Patients with diabetic foot frequently have nonpalpable pedal pulses secondary to coexistent arterial disease and medial calcinosis, and hence, bedside Doppler evaluation of pedal signals is essential to have a preliminary assessment of vascular status. 

 Visual inspection coupled with an accurate history can determine the presence of a chronic vascular condition. In chronic arterial insufficiency, the arterioles are maximally dilated as a compensatory response to the chronic ischemia intensifying color changes. In acute arterial occlusion, the venules empty, leading to a chalky white appearance regardless of extremity position. Partial but inadequate perfusion either from an incomplete acute or chronic occlusion allows for pooling of blood in the venules, which may be red in the cold or blue at higher temperatures.

 When the extremity is at the level of the heart, the pooled blood masks the color imparted by the arterial flow. Elevation of the extremity above the level of the central venous pressure (rarely >25 cm) allows the pooled venous blood to drain, enabling an accurate assessment of the degree of arterial flow. The normal extremity remains pink, whereas that with arterial insufficiency becomes pallid. Conversely, allowing the extremity to become dependent causes an intense rubor or cyanosis. The time of return of blood to the dependent extremity is a useful marker of the severity of the deficit (normally <20 seconds). With a diminished nutritional supply to the skin, there is thinning and functional loss of the dermal appendages, evident as dry, shiny, and hairless skin. The nails may become brittle and ridged. Comparison of color and trophic changes between extremities gives a good indication of the severity of the process unless a bilateral deficit is present, in which case the experience of the examiner is required to make an accurate diagnosis.

 Skin temperature is a reliable indicator of the blood flow rate in the dermal vessels, though flow is governed primarily by constriction or dilation of the arterioles to maintain a constant core temperature. Nevertheless, the temperature of the skin as a marker of perfusion is useful and can be assessed by lightly palpating the skin with the back of the hand and comparing similar sites from one extremity to the other. An ischemic limb is cool, and demarcation of temperature gives a rough indication of the level of the occlusion. Again, assessment of temperature differences is confounded when both extremities are affected.

### 4.3. Ulcer Evaluation

 Specific characteristics of the ulcer such as location, size, depth, and appearance should be recorded during the initial evaluation and with each subsequent follow-up visit to record progress and evaluate the treatment regimen [38]. Ulcers of the foot should be gently examined with a cotton-tipped probe to establish the presence of a sinus tract. The margins of the ulcer should be undermined to evaluate the extent of tissue destruction. Ulcer extension to tendon, bone, or joint should be sought. A positive probe-to-bone finding has a high predictive value for osteomyelitis and is an extremely sensitive and cost-effective screen [39]. In a study involving 132 consecutive patients, Lozano et al. demonstrated that probe-to-bone test has a sensitivity of 98%, specificity of 78%, positive predictive value of 95%, and negative predictive value of 91% (*P* < 0.001) when compared to gold standard of bone histology and culture [40]. Other, although less accurate, indicators of coexisting osteomyelitis in a diabetic foot ulcer include clinical signs of infection, radiography signs of osteomyelitis, and ulcer specimen culture. 

 Ulcerations caused by ischemia are typically located on the tips of the toes and between the digits. The lesions often appear punched out and are painful but exhibit little bleeding. Ischemic ulcers are characterized by absence of bleeding, pain, and a precipitating trauma or underlying foot deformity. They also often develop on the dorsum of the foot and over the first and fifth metatarsal heads. Ischemic ulcers are uncommon on the plantar surface as the pressure is usually less sustained, and the perfusion is better. A heel ulcer can develop from constant pressure applied while the heel is in a dependent position or during prolonged immobilization and bed rest. It should not be a surprise that a patient with relatively mild symptoms of arterial insufficiency develops limb-threatening extremity ulcers. This is due to the fact that once an ulcer is present, the blood supply necessary to heal the wound is greater than that needed to maintain intact skin. A chronic ulcer will develop unless the blood supply is improved.

 Neuropathic ulcerations typically occur at the heel or over the metatarsal heads on the plantar surface at pressure points (mal perforans ulcer) but may also occur in less characteristic locations secondary to trauma. They usually are painless. The sensory neuropathy in the diabetic patient may allow the destructive process to go unchecked, with extension into the deep plantar space and minimal appreciation by the patient.

 In addition to ulcers, patients may present with varying degrees of tissue loss or frankly gangrenous digits, forefoot, or hindfoot. The presence of dry gangrene is a relatively stable process allowing for a complete vascular evaluation; however, any progression to an infected wet gangrene requires immediate surgical debridement.

 Imaging techniques can be used to diagnose osteomyelitis and confirm the presence of bony deformities. Plain film radiography is used primarily to exclude bony lesions as a cause of a patient's pain complaints, assess the presence of osteomyelitis beneath a ulcerated foot lesion, and assess the degree of vascular wall calcification (usually in concert with standard IV contrast angiography). Plain films of the foot are relatively inexpensive and can show soft-tissue swelling, disruption of bone cortex, and periosteal elevation. MRI can provide details of pathologic anatomic features and has a high sensitivity for assessment of deep space infection and the presence of osteomyelitis in the diabetic foot.

### 4.4. Vascular Testing

 A handheld Doppler ultrasound should be used in case of inability to easily palpate a given vessel. These can be supplemented with noninvasive vascular tests and other diagnostic tests as necessary for each clinical situation. An ankle-brachial index is an important tool for assessing perfusion to the foot. Patients with an ABI less than 0.6 often experience claudication; patients with an ABI less than 0.3 may complain of rest pain; in patients with tissue loss, the ABI is often less than 0.5 [36]. In patients with diabetes and renal failure due to calcification of the vessel, ABI may be falsely elevated and is not reliable to evaluate the level of ischemia. Toe-brachial index, measured by placing small cuffs on toes, is a better indicator of foot perfusion in patients with diabetes due to the fact that toe vessels are relatively spared from the atherosclerotic disease process. 

 Duplex ultrasound is an integral component of diagnostic testing for the evaluation and management of arterial disease. This technology combines the acquisition of blood flow (pulsed Doppler spectral analysis) and anatomic (B-mode and color Doppler imaging) information. Contemporary duplex ultrasound systems provide high-resolution B-mode ultrasound imaging of tissue and vessel anatomy, including three-dimensional vessel reconstruction and evaluation of atherosclerotic plaque morphology. The duplex testing performed in the vascular laboratory is an extension of clinical assessment and is used to verify the presence and extent of disease, the involved arterial segment, and its severity. In selected patients, duplex testing can obviate the need for diagnostic arteriography for decisions regarding suitability for endovascular intervention or bypass grafting. For the diagnosis of stenosis or occlusion involving the femoropopliteal artery segment, diagnostic accuracy exceeds 95% [41]. Other noninvasive imaging methods useful in the assessment of patients with leg ulcers include plain radiography, MRI, MR angiography, and CT angiography [42].

 The assessment of a patient with foot ulcers stemming from peripheral vascular disease encompasses a thorough history and physical examination with the adjunctive use of the noninvasive vascular laboratory to confirm, localize, and grade lesions [36]. Precise, comprehensive anatomic imaging is the cornerstone of successful revascularization of the ischemic lower extremity in patients with diabetes mellitus. Contrast arteriography has been the mainstay for many years and remains the gold standard due to its superior image resolution and being the only modality used for both diagnosis and treatment. Coexistent renal insufficiency, however, makes conventional angiography impractical in significant percent of diabetic patients. CO_2_ angiogram and noninvasive Doppler studies are other alternative imaging options in this patient population. While multiple noninvasive and invasive methods are available to assess the peripheral vasculature, it should be obvious that not every patient requires an exhaustive battery of tests in order to evaluate his or her vascular status. In general, only those tests likely to provide information that alters the course of action should be performed. Differing clinical syndromes mandate the extent of peripheral vascular testing. It is imperative that flow-limiting arterial lesions are evaluated and reconstructed or bypassed if ischemic foot ulcers are to heal.

### 4.5. Neurologic Testing

 The lower extremity neurologic examination is essential and should include testing for motor strength; deep-tendon reflexes; vibratory, proprioceptive, and protective sensation [43]. Loss of protective sensation due to peripheral neuropathy is the most common cause of ulceration in the diabetic population. The use of monofilament gauges (Semmes-Weinstein) is a good objective way of assessing diabetic neuropathy [43]. Patients with normal foot sensation usually can feel a 4.17 monofilament (equivalent to 1 g of linear pressure). Patients who cannot detect a 5.07 monofilament when it buckles (equivalent to 10 g of linear pressure) are considered to have lost protective sensation [44, 45]. Several cross-sectional studies have indicated that foot ulceration is strongly associated with elevated cutaneous pressure perception thresholds [43, 46]. Magnitudes of association, however, were provided in a case-control study, where an unadjusted sevenfold risk of ulceration was observed in those patients (97% male) with insensitivity to the 5.07 monofilament [47].

 Screening is vital in identifying diabetic neuropathy early, thus enabling earlier intervention and management to reduce the risk of ulceration and lower extremity amputation. Although the nerve conduction test is the gold standard, its expense and limited availability prevent its widespread application as a screening tool for diabetic neuropathy. Semmes-Weinstein monofilament is a convenient, inexpensive, and painless alternative to NCS that should be utilized in the initial evaluation of all patients with diabetes mellitus as a screen for peripheral neuropathy. A positive Semmes-Weinstein monofilament result is a significant predictor of future ulceration and likely lower extremity amputation as well in patients with diabetes mellitus [46]. If diabetic patients have positive monofilament results, their chances of ulceration increase with 10% to 20%, corresponding with a 2.5 to 5 times higher risk than patients with normal sensation as determined by monofilament. Additionally, the risks of leg amputation increase 5% to 15%, which corresponds with a 1.5 to 15 times higher risk for patients with diabetes mellitus with positive monofilament results compared with those with negative monofilament results. The Semmes-Weinstein monofilament is an important evidence-based tool for determining which patients are at increased risk of complications during followup, leading to improved patient selection for early intervention and management. Ultimately, screening with Semmes-Weinstein monofilament may lead to improved clinical outcomes for patients with diabetic foot [46].

 The presence of neuropathy mandates attention to the biomechanics of the foot. The role of the podiatrist or podiatric foot and ankle surgeon in the evaluation of these patients cannot be underscored enough [48]. Use of a computerized gait analysis system to assess abnormally high pressure areas has led to greater use of orthotic devices in the prevention of skin breakdown. For example, an F scan system uses an ultrathin Tekscan sensor consisting of 960 sensor cells (5 mm^2^ each). The sensor is used in a floor mat system designed to measure barefoot or stocking-foot dynamic plantar pressures, indicating those subjects with pressures greater than or equal to 6 kg/cm^2^. Abnormal mechanical forces that can result in ulcerations should be addressed with the use of offloading devices or other modalities in order to assist in wound healing.

 Particular attention should be paid to documenting a complete neurologic examination on patients who have suffered from a previous stroke, as much of the rationale for extremity salvage hinges on the potential for rehabilitation. The remainder of the physical examination should be undertaken with attention to the presence of comorbidities, which may influence the decision making process.

## 5. Management of Diabetic Foot Ulcers

### 5.1. General Medical

 People with diabetes should receive close metabolic management preferably from health professionals with expertise and a special interest in diabetes. The primary techniques available to assess the effectiveness of the management plan on glycemic control are patient self-monitoring of blood glucose and hemoglobin A1C levels. Since A1C levels are thought to reflect average glycemia over several months and have strong predictive value for diabetes complications, A1C testing should be performed routinely in all patients with diabetes, at initial assessment and then as part of continuing care [49]. Lowering A1C to below or around 7% has been shown to reduce microvascular and neuropathic complications of diabetes and, if implemented soon after the diagnosis of diabetes, is associated with long-term reduction in macrovascular disease [50]. 

### 5.2. Vascular

 Management of ischemic ulcers follows some basic guiding principles. It is imperative that flow-limiting arterial lesions be evaluated and reconstructed or bypassed [51]. In general, the optimal strategy is to perform revascularization, if indicated, as soon as possible. Closure of the ulcer by primary healing or secondary reconstructive surgery will then be expedited. If revascularization of an ischemic ulcer is not possible for medical or technical reasons, amputation of the foot or limb will most likely result. Contraindications to revascularization include nonambulatory patients and a foot phlegmon with sepsis or excessive foot gangrene, precluding a functional foot despite adjunctive plastic surgical procedures such as skin grafts and free flaps. Nonoperative management of patients with lower extremity ischemia consists of general wound care measures. As a rule, however, severe ischemia of the lower limb generally requires an interventional approach. The method of revascularization of the affected limb depends on several factors, among the most important being the indications for surgery, the patient's operative risk, arteriographic findings, and available graft material.

 The impact of increasing numbers of revascularization procedures on the rate of amputation in patients with vascular disease remains a current source of continuing debate. In the 2007 update to the Trans-Atlantic Inter-Society Consensus Document on Management of Peripheral Artery Disease (TASC II), older US studies cited had shown no reduction on amputation rates by increasing revascularization procedures [52]. Nonetheless, more recent data from Sweden, Denmark, and Finland reflected a significant decrease in amputations with the increased availability and use of both endovascular interventions and surgical revascularization [52]. UK data demonstrated a plateau in major amputations that may reflect increasingly successful limb salvage [52]. Recent studies of Medicare B claims between 1996–2006 confirmed trends similar to the European studies, that is, a clear decrease in total lower extremity amputations with increasing numbers of vascular interventions [53]. 

 The long-held gold standard in vascular surgery for lower extremity revascularization procedures is the performance of an arterial bypass with autologous saphenous vein graft. A twenty-year review of infrainguinal revascularization procedures published in 2011 [54] reaffirmed the superiority of saphenous vein as the bypass material of choice in all positions over time, regardless of the inflow or outflow vessel; 5- and 10-year patency for femoropopliteal bypass, the most common procedure, was 83% and 63%, respectively, with an associated limb salvage rate of 89% over the same period. A recent trend that emerged over the study period was the increased use of polytetrafluoroethylene as the conduit material. While the 5- and 10-year patency rates (62% and 24%) and limb salvage rates (71% and 68%) were inferior to vein, this synthetic material allowed the expanded availability of bypass surgery to patients who previously had no surgical option due to inadequate vein for harvest, less than optimal presurgical condition, or earlier use of saphenous vein for coronary artery bypass-common comorbidities in diabetics [54].

 While the most durable and effective revascularization procedure is surgical bypass, endovascular angioplasty stenting provides a less invasive alternative in indicated patients. Specifically, patients with short-segment disease in proximal locations, such as isolated iliac artery stenoses, are prime candidates for angioplasty and endovascular stent placement [55]. While many trials have shown poorer long-term patency rates with more frequent interventions when endovascular interventions are compared to open surgery, the BASIL trial, a large multicenter UK study, found that limb salvage rates were similar between balloon angioplasty and bypass surgery [56]. Unfortunately, angioplasty has been disappointing in dealing with complex infrainguinal disease.

 Though it was first described in the 1990s, the endovascular technique of subintimal angioplasty has become established recently in the treatment of lower extremity arterial occlusions. Conceptually similar to an endovascular bypass procedure, the procedure consists of creating a dissection in the subintimal plane to cross an occluded arterial segment, then to re-enter the patent distal true arterial lumen. This dissection plane is expanded with an angioplasty balloon, thus creating a nonanatomic bypass canal free of atheromatous plaque [57]. Meta-analyses on subintimal angioplasty outcomes has shown results at the 12-month timeframe of a primary patency of 55.8%, clinical success rates of 50%–70%, with an associated limb salvage rate of 80%–90%. Limb salvage rates at 4 years, however, were noted to be as low as 34% [58, 59]. The adoption of this technique has been able to expand the scope of therapy to those with complex infrapopliteal occlusions and those patients considered to have a high cardiac risk who are otherwise unsuited for open surgical repair. 

 Another emergent technological advancement in interventional therapy has been the addition of endovascular atherectomy to the vascular surgeon's arsenal. The method by which atherectomy—the removal of atherosclerotic lesions from the artery—is undertaken is based highly on the system being used by the surgeon. Options include ablative lasers, which can be associated with thermal damage complications, and excisional modalities to strip or grind the atherosclerotic plaque from the arterial walls, with the potential to create embolic complications downstream from the affected area [60]. The theoretical advantages of atherectomy over traditional angioplasty and stenting are in lesion debulking and minimizing barotrauma to the artery. Eliminating this stretch injury on the arterial walls has the potential to reduce the rate of restenosis by minimizing a known stimulant for neointimal hyperplasia [61]. 

While not a novel recent technique, renewed interest in interventional circles has been generated with the introduction of new technology [60]. The rapid evolution of this technology can be reflected in the fact that the FDA has approved four new atherectomy devices for the treatment of peripheral arterial disease over the last decade [62]. Previous iterations of these devices, however, have not demonstrated any significant long-term benefit over angioplasty; it remains to be seen whether the new generation of devices shows additional clinical benefit.

### 5.3. Wound Care

 Aggressive mechanical debridement, systemic antibiotic therapy, and strict nonweight bearing are the cornerstones for effective wound care [63]. The role of a multidisciplinary group of consultants in the management of diabetic ulcers cannot be overemphasized [64]. Successful management of foot ulcers involves recognition and correction of the underlying etiology, as well as appropriate wound care and prevention of recurrence. Sharp debridement in the operating room or at the bedside, when applicable, allows for thorough removal of all necrotic material and optimizes the wound environment [65]. While surgical debridement is swift and effective, it requires a skilled operator to carry it out safely and thoroughly. Debridement using a scalpel produces a very clean wound, but healthy collateral tissue around the wound is also removed. Nonsurgical methods of debridement include autolysis—often facilitated by dressings; larval therapy using sterile maggots; chemical debridement using collagenases or papain-urea ointments. These are all effective in certain wounds but tend to be slow, and sometimes unpredictable. Advanced debridement techniques like hydrosurgery and ultrasonic debridement can be helpful in eliminating necrotic tissue in preparation for wound closure. Recent developments in hydrosurgery provide more control over the surgical debridement process. One system uses pressurized saline in a sterile circuit that is forced into a nozzle [66]. The water executes a 180° turn and is forced out of a miniscule nozzle, less than 0.0005 inch diameter, where it emerges as a focused jet. The water jet passes parallel to the wound and is captured by an evacuator port creating a Venturi effect. Venturi effect associated with the flow carries the water jet, ablated tissue, and debris into the evacuator port without the need for separate suction. The debridement therapy is clearly visible without any accompanying thermal damage to the tissue. There is minimal bleeding with excellent preservation of the healthy collateral tissue. 

 All necrotic bone and devascularized tissues, plus a small portion of the uninvolved bone and soft tissue, should be excised to establish the degree of penetration of the infection [65] Curettage of any exposed or remaining cartilage is important to prevent this avascular structure from becoming a nidus of infection. Foot soaks, whirlpool therapy, or enzymatic debridement have a use but are rarely effective and may lead to further skin maceration or wound breakdown. No prospective randomized studies have demonstrated the superiority of dressing products compared with standard saline wet to dry sterile gauze in establishing a granulation bed. Use of moist dressings in clean, granulating wounds is recommended to enhance the wound environment [67, 68]. An “ideal” dressing not only provides protection against further bacterial contamination but also maintains moisture balance, optimizes the wound pH, absorbs fibrinous fluids, and reduces local pain. Many advanced moist wound therapies are available, and each has its own distinct characteristics. Hydrogels, Hydrocolloids, Alginates, and many silver compounds have been found to promote wound healing and reduce bacterial contamination [69]. Various dressings are currently available to target specific characteristics of the wound; however, moist normal-saline dressings are probably sufficient for most wounds [70].

### 5.4. Control of Infection

 Infection control is paramount to the success of wound conversion. The absence of systemic manifestations such as fever, chills, or leukocytosis is an unreliable indicator of underlying infection, especially in the diabetic immune-compromised population. Systemic antibiotics must be given as early as possible in cases of clinically infected diabetic foot ulcers, and the use of topical antibiotics and antiseptics is not recommended as the sole treatment of infection. In cases of gross wound infections and rampant cellulitis, use of a silver-containing medication may be necessary in the initial setting to reduce the bacterial load. Oral antimicrobial therapy should be instituted on the basis of the suspected pathogen and clinical findings. IV antimicrobials should be administered for severe infections. 

 Polymicrobial infections are common in the diabetic foot, but the majority of pathogens remain Gram positive [28]. These patients require immediate hospitalization, broad-spectrum IV antibiotics, and aggressive surgical debridement. Superficial wound cultures are often unreliable, as they may demonstrate organisms responsible for colonization that do not affect the associated infection. Deep wound or bone cultures are the best way to accurately assess the microbiology in a diabetic foot infection and to assess for osteomyelitis [27]. Severe infections, however, should be treated with broad-spectrum IV antibiotics with particular emphasis on the role of biofilms [30, 34]. Initial broad spectrum systemic therapy is continued until adequate cultures are available. A severity grading of the wound can assist in choice of antibiotics. The antibiotic regimen must always include an agent active against Gram-positive cocci, particularly *S. aureus*. Other factors to be considered when selecting an appropriate antibiotic combination and the route of administration include severity of infection, previous allergy or intolerance, patient compliance, renal and/or hepatic dysfunction, peripheral arterial disease and any devitalization of the tissues surrounding the wound,recent exposure to antibiotic therapy or hospital admission, chronicity of the wound, knowledge of local potential pathogens, and antibiotic sensitivity patterns severity of infection. IDSA guidelines have been developed as a tool for determining appropriate antimicrobial therapies.

 The emergence of resistance poses many unique problems in reference to diabetic foot infections. MRSA, VRE, and Gram-negative resistance have created new challenges for those caring for the diabetic foot [29]. Development of newer antibiotic therapies for resistant pathogens is timely [32]. However, as emphasized by Nelson et al. and recently confirmed in a systematic review, no one particular antimicrobial agent or regimen has yet been shown to be superior to others in curing diabetic foot infection [33]. Once antibiotic treatment is initiated, the wound must be regularly and carefully inspected to assess the response to therapy. Once microbiological culture and sensitivity testing results are available, the initial regimen should be adjusted to use the most effective narrow-spectrum regimen. The optimal duration of antibiotic treatment is not clearly defined and depends on severity of infection and response to treatment. Most authorities suggest one- to two-week antibiotic therapy for mild infections, whereas treatment must be extended for up to 1 month or more for more severe infections.

### 5.5. Wound Closure

 After bacterial contamination has been controlled, small ulcers can usually be excised and closed immediately. Use of local rotation flap helps in primary closure of the excised wounds without any undue tension on the suture line. This allows the use of local tissue, which is more suited for weight bearing, for wound closure. Large open wounds, however, are treated with a staged approach, with frequent debridement and establishment of a granulation base. The clean wounds can then be closed with healthy tissue, with the use of local or free-flap coverage and soft-tissue repair. Meticulous surgical reconstruction of these wounds can help avert the production of inelastic scar tissue over weight-bearing surfaces. Any remaining extrinsic or intrinsic pressures can be reduced with the postoperative use of orthoses.

 The endpoint for chronic diabetic foot wounds should include reduction in the number of major amputations, prevention of infection, decreased probability of ulceration, maintenance of skin integrity, and improvement of function. Reconstructive foot surgery plays a vital role in avoiding major amputations in these chronic neuropathic wounds. Successful outcomes for diabetic foot reconstruction should result in less intrinsic pressures via minor amputations, arthroplasties, osteotomies, condylectomies, exostosectomies, tendon procedures, and joint arthrodesis [71]. Open wounds can be treated in one stage and are primarily closed with premorbid tissue using local flap reconstruction and soft tissue repair [31]. Plastic surgical repair of these wounds can help avoid the production of inelastic scar tissue over weight-bearing surfaces [72]. Extrinsic and intrinsic pressures can be further neutralized with postoperative accommodative shoe gear [73, 74]. Prophylactic diabetic foot surgery is an increasingly used option to prevent recurrent ulceration and reduce the risk of major amputations [75, 76]. Surgical biomechanics, plastic and soft tissue reconstruction, and appropriate offloading are all essential to create a stable platform from which to keep these difficult patients free from tissue breakdown and as functional as possible.

 Treatment of these pedal soft-tissue deficits in the diabetic patient population continues to be a medical and surgical challenge, which extends the length of the patient's disability and significantly increases the cost of medical care. Simple closure of these wounds is often difficult because of preexisting bone deformity, tissue inelasticity, location of the defect, and superimposed osteomyelitis.

### 5.6. Off-Loading

 Off-loading strategies such as total contact casting or removable walkers have resulted in significant decreases in healing times [73, 74]. The stresses placed on the foot can be intrinsic, as was previously described with respect to digital contractures, or extrinsic in nature. These external forces can result from inappropriate footwear, traumatic injury, or foreign bodies. Shoes that are too tight or too shallow are a frequent yet preventable component to the development of neuropathic ulcers. Various shoe modifications such as the rocker-sole design and different types of insoles have made it possible to reduce plantar foot pressures, thus decreasing the risks of ulceration [77–79]. 

### 5.7. Negative Pressure Wound Therapy

Management of even the most superficial wounds in a diabetic foot is difficult with poor healing responses and high rates of complications. Although several advanced debridement and dressing techniques have been developed to improve wound healing, achieving adequate wound closure is a major problem. Negative pressure wound therapy (NPWT) has emerged as an effective treatment for these complex wounds [71]. This involves application of subatmospheric pressure to the wound through open-celled foam dressing in a closed environment. The pump is connected to a canister, which collects the wound exudates. Besides the convenience of wound care, NPWT has been shown to stimulate angiogenesis, increase rate of granulation tissue, and decrease bacterial colonization while decreasing edema and increasing blood flow [80]. Multiple studies have indicated that NPWT is a safe and effective treatment for complex diabetic foot wounds, and could lead to a higher proportion of healed wounds, faster healing rates, and potentially fewer reamputations than standard care. When compared to advanced moist wound therapy in a multicenter randomized control trial, NPWT achieved wound closure in 43% patients as opposed to 29% patients being treated by conventional techniques (*P* = 0.007). Patients with NPWT also experienced fewer secondary amputations and a significantly shorter healing time [81].

 Once healthy granulation of deep complex wounds is achieved, restoration of intact skin barrier is of utmost importance to prevent infection, minimize wound contraction to maintain function, minimize cosmetic disfigurement, and to avoid volume depletion. Traditionally, split-thickness skin grafts (STSGs) are used to cover large areas of skin loss, granulating tissue beds, and tissue loss across joints in areas where contraction will cause deformity and where epithelialization alone will produce an unstable wound cover. STSGs currently represent the most rapid, effective method of reconstructing large skin defects. The conventional therapy dressing of choice, to secure the graft while it is healing, is a cotton bolster or sterile compressive or stainless steel gauze dressing that is used for at least five days. NPWT has been increasingly used as an alternative dressing following STSG and has achieved improved graft survival while reducing the incidence of complications like seroma, hematoma, and infection. Mechanisms of action of NPWT include reducing edema from extracellular tissues, decreasing the bacterial load on the wound, and promoting tissue perfusion and healthy granulation tissue formation [82–84]. 

### 5.8. Alternative and Adjunctive Therapies

 Even when properly managed, the wounds may not heal in a timely fashion. Foot ulcers that do not heal in an expedient amount of time are expected to be more likely to become complicated by intervening infection, hospitalization, and amputation and, thus, to be more costly because of the increased utilization of healthcare resources. Therapists generally rely on good clinical judgment and personal experience in deciding when to use more aggressive or more expensive technologies and interventions. In a prospective randomized controlled trial in 203 patients, wound area reduction of greater than 52%, both absolute and relative, over a 4-week period, was a strong predictor of complete wound healing over an extended 12-week period (58% healing rate versus 9% healing rate) [85]. 

 Many agents have been suggested to be used as adjuvants, to aid healing, in the treatment of diabetic ulcers. These therapies include topical agents for application to the wound bed (e.g., Recombinant PDGF, Regranex), systemic therapies (hyperbaric oxygen) to treat the patient, and skin substitutes (e.g., Apligraf, Dermagraft). These agents have shown promising results and have proven useful under specific circumstances. There is level I evidence that platelet-derived growth factor (PDGF) is effective in treating diabetic neurotrophic foot ulcers. PDGF is a powerful chemoattractant and mitogen, exerting its action on fibroblasts, smooth muscle cells, and endothelial cells. It also induces production of fibronectin and hyaluronic acid. Margolis and colleagues examined the effectiveness of recombinant PDGF (becaplermin) in 24,898 subjects with neuropathic foot ulceration. Healing rates were 33.5% and 25.8% in the becaplermin and control group, respectively, (*P* < 0.0001) consistent with increased likelihood of healing by 32%. Moreover, amputation rates were significantly (*P* < 0.0001) lower in the becaplermin (4.9%) than in the control group (6.4%). [86]. Other cytokine growth factors do not yet have enough data on efficacy to recommend any of them for treatment of diabetic ulcers, although isolated reports suggest their potential usefulness [87]. 

 Tissue-engineered skin (Apligraf, Organogenesis) comprises a cultured living dermis and sequentially cultured epidermis, the cellular components of which are derived from neonatal foreskin. In a randomized trial involving 208 patients, the rate of healing at 12 weeks was higher among those who used tissue-engineered skin (applied weekly for up to 5 weeks) and received good wound care (debridement and elimination of pressure) than among those who received good wound care alone (56 percent versus 38 percent, *P* = 0.004). Treatment with tissue-engineered skin was associated with faster healing and lower rates of osteomyelitis (3 percent versus 10 percent in the control group; *P* = 0.04) and lower-limb amputation (6 percent versus 16 percent, *P* = 0.03) [88]. The failure to reduce the size of an ulcer after four weeks of treatment that includes appropriate debridement and pressure reduction should prompt consideration of adjuvant therapy. Adjunctive therapies are in general limited due to a combination of their substantial costs and poor reproduction of results of controlled clinical trials in actual clinical practice [89]. Other investigational adjuvant therapies for diabetic foot include electrical stimulation of the ulcer bed, therapeutic ultrasound, application of electromagnetic fields, and therapeutic heat.

### 5.9. The Reconstructive Ladder

Reconstructive surgery can range from simple metatarsal head resections to subtotal calcanectomies. Local flaps that are often difficult to elevate and inset are more easily mobilized and incised when concomitant bone resection is achieved at the time of flap creation. In addition, a local flap results in greater exposure and direct visualization of the underlying osseous structures compared with a single linear or semielliptical incision. The implementation of local random flaps can eliminate the need for additional incisions often deemed necessary to gain access to a forefoot, midfoot, or rearfoot bony defect. The use of negative pressure wound therapy has greatly enabled the salvage of these complex limb wounds [71, 81].

## 6. Prevention of Recurrence of Diabetic Foot Ulcers

 Diabetic ulcers of lower extremity are a chronic problem with recurrence rates of 8%–59%. Therefore, long-term maintenance must be addressed even for healed ulcers. This includes identification of high-risk patients, education of the patient, and institution of measures to prevent ulceration. High-risk patients should be identified during the routine foot examination performed on all patients with DM. Patient education should emphasize careful selection of footwear, daily inspection of the feet to detect early signs of poor-fitting footwear or minor trauma, daily foot hygiene to keep the skin clean and moist, avoidance of self-treatment of foot abnormalities and high-risk behavior (e.g., walking barefoot), and prompt consultation with a health care provider if an abnormality arises. Any diabetic patient admitted to acute care setting should have their feet examined on admission. If it is judged that their feet are at risk of new ulceration, preventive steps should be taken immediately, which includes provision of a pressure mattress and suitable protective footwear. Those with new ulcers should be referred promptly to an expert multidisciplinary team for expert assessment and management [90].

 There is strong evidence that introduction of a specialist podiatry service and a comprehensive diabetes education and care management program in the dialysis unit results in a prompt decline in the incidence of amputation. McMurray and McDougall demonstrated that introduction of such a program in dialysis unit results in significant stabilization of the diabetes-related peripheral vascular/neuropathic disease and the 12 month foot risk assessment score. While there was no difference in the mortality, the study group had a statistically significant lower hospitalization rate for diabetes, peripheral vascular, infection, and amputation-related admissions (*P* < 0.05) [91].

## 7. Socioeconomic Issues of Diabetic Foot Ulceration

 The median time of healing for a diabetic foot ulcer is approximately six months. During this time, most of patients require multiple hospitalizations to health care facilities for reasons like infection control, debridement, wound closure, revascularization, and other medical complications. Ulceration and infection of lower extremities are the leading causes of hospitalization in patients with diabetes [92]. Treatment of pedal soft-tissue deficits in the diabetic patient population continues to be a medical and surgical challenge, extending the length of their disability and significantly increasing the cost of medical care. Despite all interventions, only two thirds of ulcers eventually heal with the remainder resulting in some form of amputation. In 2005, approximately 1.6 million people were living with limb loss and this number is expected to more than double by 2050 [13]. Worldwide, over one million lower extremity amputations are performed annually on people suffering from diabetes, and the majority of these amputations are preceded by ulcers. Nearly half of all patients who undergo amputation will develop limb-threatening ischemia in the contralateral limb, and many will require an amputation of the opposite limb within five years. In 2000, the Centers for Disease Control and Prevention (CDC) released the national diabetes fact sheet which estimated that 12 million Americans were diagnosed with diabetes each year with one in five diabetes dollars spent on lower extremity care.For the year 2011, the CDC estimated a total of 25.8 million Americans diagnosed with diabetes with an estimated prevalence of 8.3%. The total cost of diagnosed diabetes in 2007 was $174 billion, of which $116 billion was spent as direct medical costs and $58 billion on indirect costs attributed to disability, loss of productivity, and premature mortality. In the year 2010, about 1.9 million new cases of diabetes were added to the existing pool in the age group of 20 years and older [1]. In 2007, diabetes contributed to a total of 231,404 deaths. More than 60% of nontraumatic lower-limb amputations occur in people with diabetes, which amounted to 65,700 nontraumatic lower-extremity amputations in 2006. 

 The average cost of diabetic foot ulcer treatment ranges from $3609 to $27,721 [93]. As expected, the cost of diabetic foot ulcer care increases as the severity of the wound increases. The average diabetic foot ulcer cost has been reported to be approximately four times higher in patients with peripheral arterial disease ($23,372) compared with patients with neuropathic wounds ($5218). The greatest expense for diabetic foot ulcers is for therapies that are not effective, because patients with unhealed wounds are more likely to have bone and soft-tissue infections that require hospitalizations [94]. In England, the estimated cost of care for patients with leg ulcers in a population of 250,000 was about $130,000 annually per patient [95]. Items factored into the equation include physician visits, hospital admissions, home health care, wound care supplies, rehabilitation, time lost from work, and jobs lost. Adding to the cost is the chronic nature of these wounds, the high rate of recurrence, and the propensity to become infected.

 Preventing ulcerations and/or amputations is critical from both medical and economical standpoints. Due to the fact that chronic ulceration affects a patient's lifestyle and mobility, leg ulcers carry an enormous social cost. One of the indicators of this social cost is health-related quality of life index (HRQOL) which is defined as the sum of the physical, emotional, and social issues in a person's life that may be affected by, or may affect, a health issue. HRQOL may include factors such as physical health, pain, mobility, emotional state, dependence on others, difficulty with usual activities, and living conditions. HRQOL is worse among individuals with diabetes than in individuals without diabetes. There are multiple variables that are associated with a poorer HRQOL in patients with diabetes. There is substantial evidence, however, that the most important variable affecting HRQOL of people with diabetes is the presence of complications of which diabetic foot ulcer is the major factor [96–98]. Diabetic foot ulcers result in significant decrements in quality of life, including decreased mobility, falls, increased dependence on others, loss of employment, reduced income, increased risk of amputation, repetitive trips to the physician or clinic for care, and increased expense. The negative effect of diabetic foot ulcers on HRQOL results in large part from reduced mobility. The loss of mobility directly affects the individual's ability to engage in common everyday tasks and to participate in leisure activity. Deficits related to activities of daily living may also compromise HRQOL. Compromised mobility and the need to keep the foot dressing dry may limit self-care activities, such as bathing, and patients report loss of self-esteem related to altered hygiene patterns. The ability to work may be temporarily or permanently affected by the condition [10]. Employment is often markedly affected by the presence of the diabetic foot ulcer or associated treatment, and financial hardship is a major issue for many patients. The majority (50%–79%) of patients with diabetic foot ulcers are unemployed, have retired early, or are unable to work because of the ulcer [99]. Conservative estimates indicate that about 10 million workdays are lost in the United States annually secondary to lower extremity ulcers [100, 101]. A report in 1994 focused on the financial, social, and psychological implications of lower extremity lesions in 73 patients [102]. Among the study patients, 68% reported feelings of fear, social isolation, anger, depression, and negative self-image because of the ulcers. In addition, 81% of the patients felt that their mobility was adversely affected. Within the younger actively working population, there was a strong correlation between lower extremity ulceration and adverse effect on finances, time lost from work, and job loss. In addition, there was a strong correlation between time spent on ulcer care and feelings of anger and resentment. 

## 8. Summary

**Figure 6:**
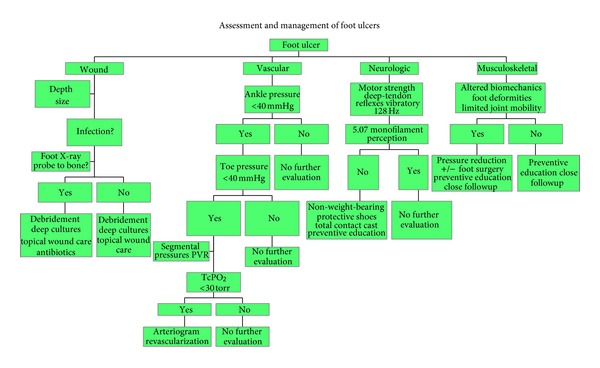
Sample algorithm for assessment and management of diabetic foot ulcers. The management of the diabetic foot is a complex clinical problem that requires multidisciplinary inputs.

 Chronic diabetic foot ulcers are frequently encountered in clinical practice. The cost of chronic nonhealing wounds is enormous and is accompanied by considerable morbidity and mortality. Careful assessment of vascular disease, evaluation and management of biomechanical and metabolic abnormalities and aggressive treatment of any infections are required. Surgical correction of biomechanical defects, plastic and soft-tissue reconstruction, and appropriate measures to minimize foot pressure are all essential to enable the patient to walk effectively again. Likewise, the use of negative pressure wound therapy has been a big advance in the care of advanced wounds [80, 81, 103]. Clinical pathways related to diabetic foot ulcers frequently involve persistent sharp debridement, expensive wound care products, long-term IV antibiotics, total contact casting with tendo-Achilles lengthening, use of skin equivalents, electrical stimulation, multiple off-loading orthopedic devices, and even amputation. The multidisciplinary approach provides a comprehensive treatment protocol and significantly increases the chances of successfully healing the ulcer and preventing recurrence, as sample algorithm shown in Figure 6.

 Despite being one of the most serious and costly complications of diabetes, foot complications can be effectively prevented. By implementing a care strategy that combines prevention, multidisciplinary treatment of foot ulcers, appropriate organization, close monitoring, and education of both healthcare professionals and people with diabetes, it is possible to reduce amputation rates by up to 85%.

## References

[2] American Diabetes Association (1999). Preventive foot care in people with diabetes [position statement]. *Diabetes Care*.

[3] Reiber GE, Lipsky BA, Gibbons, W G (1998). The burden of diabetic foot ulcers. *American Journal of Surgery*.

[4] Reiber GE (1996). The epidemiology of diabetic foot problems. *Diabetic Medicine*.

[5] Knox RC, Dutch W, Blume P, Sumpio BE (2000). Diabetic foot disease. *International Journal of Angiology*.

[6] Murray HJ, Boulton AJM (1995). The pathophysiology of diabetic foot ulceration. *Clinics in Podiatric Medicine and Surgery*.

[7] Hutton W, Stokes I, Klenerman L (1991). The mechanics of the foot. *The Foot and Its Disorders*.

[8] Habershaw G, Chzran J (1995). Biomechanical considerations of the diabetic foot. *Management of Diabetic Foot Problems*.

[9] Sumpio BE (2000). Foot ulcers. *New England Journal of Medicine*.

[10] Phillips TJ (1994). Chronic cutaneous ulcers: etiology and epidemiology. *Journal of Investigative Dermatology*.

[11] O’Brien IAD, Corrall RJM, Krolewski AS (1988). Epidemiology of diabetes and its complications. *New England Journal of Medicine*.

[12] Laing P (1998). The development and complications of diabetic foot ulcers. *American Journal of Surgery*.

[13] Weiss JS, Sumpio BE (2006). Review of prevalence and outcome of vascular disease in patients with diabetes mellitus. *European Journal of Vascular and Endovascular Surgery*.

[14] Bild DE, Selby JV, Sinnock P, Browner WS, Braveman P, Showstack JA (1989). Lower-extremity amputation in people with diabetes. Epidemiology and prevention. *Diabetes Care*.

[15] Melton LJ, Macken KM, Palumbo PJ, Elveback LR (1980). Incidence and prevalence of clinical peripheral vascular disease in a population-based cohort of diabetic patients. *Diabetes Care*.

[16] Kamal K, Powell RJ, Sumpio BE (1996). The pathobiology of diabetes mellitus: implications for surgeons. *Journal of the American College of Surgeons*.

[17] Levin ME (1998). Diabetes and peripheral neuropathy. *Diabetes Care*.

[18] Boulton AJM, Hardisty CA, Betts RP (1983). Dynamic foot pressure and other studies as diagnostic and management aids in diabetic neuropathy. *Diabetes Care*.

[19] Fernando DJS, Masson EA, Veves A, Boulton AJM (1991). Relationship of limited joint mobility to abnormal foot pressures and diabetic foot ulceration. *Diabetes Care*.

[20] Lee L, Blume PA, Sumpio B (2003). Charcot joint disease in diabetes mellitus. *Annals of Vascular Surgery*.

[21] Veves A, Malik RA, Lye RH (1991). The relationship between sural nerve morphometric findings and measures of peripheral nerve function in mild diabetic neuropathy. *Diabetic Medicine*.

[22] Veves A, Fernando DJS, Walewski P, Boulton AJM (1991). A study of plantar pressures in a diabetic clinic population. *The Foot*.

[23] Masson EA, Hay EM, Stockley I, Veves A, Betts RP, Boulton AJM (1989). Abnormal foot pressures alone may not cause ulceration. *Diabetic Medicine*.

[24] Morag E, Pammer S, Boulton A, Young M, Deffner K, Cavanagh P (1997). Structural and functional aspects of the diabetic foot. *Clinical Biomechanics*.

[25] Saltzman CL, Pedowitz WJ (1999). Diabetic foot infections. *Instructional Course Lectures*.

[26] Weitz JI, Byrne J, Patrick Clagett G (1996). Diagnosis and treatment of chronic arterial insufficiency of the lower extremities: a critical review. *Circulation*.

[27] Lipsky BA, Berendt AR, Deery HG (2004). Diagnosis and treatment of diabetic foot infections. *Clinical Infectious Diseases*.

[28] Lipsky BA, Armstrong DG, Citron DM, Tice AD, Morgenstern DE, Abramson MA (2005). Ertapenem versus piperacillin/tazobactam for diabetic foot infections (SIDESTEP): prospective, randomised, controlled, double-blinded, multicentre trial. *Lancet*.

[29] Lipsky BA, Tabak YP, Johannes RS, Vo L, Hyde L, Weigelt JA (2010). Skin and soft tissue infections in hospitalised patients with diabetes: culture isolates and risk factors associated with mortality, length of stay and cost. *Diabetologia*.

[30] Davis SC, Martinez L, Kirsner R (2006). The diabetic foot: the importance of biofilms and wound bed preparation. *Current Diabetes Reports*.

[31] Blume PA, Paragas LK, Sumpio BE, Attinger CE (2002). Single-stage surgical treatment of noninfected diabetic foot ulcers. *Plastic and Reconstructive Surgery*.

[32] Citron DM, Goldstein EJC, Merriam CV, Lipsky BA, Abramson MA (2007). Bacteriology of moderate-to-severe diabetic foot infections and in vitro activity of antimicrobial agents. *Journal of Clinical Microbiology*.

[33] Nelson EA, O’Meara S, Golder S, Dalton J, Craig D, Iglesias C (2006). Systematic review of antimicrobial treatments for diabetic foot ulcers. *Diabetic Medicine*.

[34] Joshi N, Caputo GM, Weitekamp MR, Karchmer AW (1999). Infections in patients with diabetes mellitus. *New England Journal of Medicine*.

[35] Adam DJ, Naik J, Hartshorne T, Bello M, London NJM (2003). The diagnosis and management of 689 chronic leg ulcers in a single-visit assessment clinic. *European Journal of Vascular and Endovascular Surgery*.

[36] Collins KA, Sumpio BE (2000). Vascular assessment. *Clinics in Podiatric Medicine and Surgery*.

[37] Boulton AJM, Armstrong DG, Albert SF (2008). Comprehensive fool examination and risk assessment: a report of the task force of the foot care interest group of the American diabetes association, with endorsement by the American association of clinical endocrinologists. *Diabetes Care*.

[38] Pressley ZM, Foster JK, Kolm P (2007). Digital image analysis: a reliable tool in the quantitative evaluation of cutaneous lesions and beyond. *Archives of Dermatology*.

[39] Grayson ML, Gibbons GW, Balogh K, Levin E, Karchmer AW (1995). Probing to bone in infected pedal ulcers: a clinical sign of underlying osteomyelitis in diabetic patients. *Journal of the American Medical Association*.

[40] Lozano RM, González Fernández ML, Hernández DM, Beneit Montesinos JV, Jiménez SG, Gonzalez Jurado MA (2010). Validating the probe-to-bone test and other tests for diagnosing chronic osteomyelitis in the diabetic foot. *Diabetes Care*.

[41] Cossman DV, Ellison JE, Wagner WH (1989). Comparison of contrast arteriography to arterial mapping with color-flow duplex imaging in the lower extremities. *Journal of Vascular Surgery*.

[42] Sumpio BE, Lee T, Blume PA (2003). Vascular evaluation and arterial reconstruction of the diabetic foot. *Clinics in Podiatric Medicine and Surgery*.

[43] Feng Y, Schlösser FJ, Sumpio BE (2009). The Semmes Weinstein monofilament examination as a screening tool for diabetic peripheral neuropathy. *Journal of Vascular Surgery*.

[44] Armstrong DG, Lavery LA (1998). Diabetic foot ulcers: prevention, diagnosis and classification. *American Family Physician*.

[45] Birke JA, Sims DS (1986). Plantar sensory threshold in the ulcerative foot. *Leprosy Review*.

[46] Feng Y, Schlösser FJ, Sumpio BE (2011). The Semmes Weinstein monofilament examination is a significant predictor of the risk of foot ulceration and amputation in patients with diabetes mellitus. *Journal of Vascular Surgery*.

[47] McNeely MJ, Boyko EJ, Ahroni JH (1995). The independent contributions of diabetic neuropathy and vasculopathy in foot ulceration: how great are the risks?. *Diabetes Care*.

[48] Sumpio BE, Armstrong DG, Lavery LA, Andros G (2010). The role of interdisciplinary team approach in the management of the diabetic foot. A Joint Statement from the Society for Vascular Surgery and the American Podiatric Medical Association. *Journal of Vascular Surgery*.

[49] Boulton AJM (1993). DCCT: implications for diabetes care in the UK. *Diabetic Medicine*.

[50] Association AD (2011). Executive summary: standards of medical care in diabetes-2011. *Diabetes Care*.

[51] Sarage A, Yui W, Blume P, Aruny J, Sumpio B, Geroulakos G (2009). Aggressive revascularization options using cryoplasty in patients with lower extremity vascular disease. *Re-do Vascular Surgery*.

[52] Norgren L, Hiatt WR, Dormandy JA, Nehler MR, Harris KA, Fowkes FGR (2007). Inter-society consensus for the management of peripheral arterial disease (TASC II). *Journal of Vascular Surgery*.

[53] Goodney PP, Beck AW, Nagle J, Welch HG, Zwolak RM (2009). National trends in lower extremity bypass surgery, endovascular interventions, and major amputations. *Journal of Vascular Surgery*.

[54] Ziegler KR, Muto A, Eghbalieh SDD, Dardik A (2011). Basic data related to surgical infrainguinal revascularization procedures: a twenty year update. *Annals of Vascular Surgery*.

[55] Muhs BE, Gagne P, Sheehan P (2005). Peripheral arterial disease: clinical assessment and indications for revascularization in the patient with diabetes. *Current Diabetes Reports*.

[56] Adam DJ, Beard JD, Cleveland T (2005). Bypass versus angioplasty in severe ischaemia of the leg (BASIL): multicentre, randomised controlled trial. *Lancet*.

[57] Chun JY, Markose G, Bolia A (2010). Developments in subintimal angioplasty in the infrainguinal segment. *Journal of Cardiovascular Surgery*.

[58] Bown MJ, Bolia A, Sutton AJ (2009). Subintimal angioplasty: meta-analytical evidence of clinical utility. *European Journal of Vascular and Endovascular Surgery*.

[59] Met R, Van Lienden KP, Koelemay MJW, Bipat S, Legemate DA, Reekers JA (2008). Subintimal angioplasty for peripheral arterial occlusive disease: a systematic review. *CardioVascular and Interventional Radiology*.

[60] Rogers JH, Laird JR (2007). Overview of new technologies for lower extremity revascularization. *Circulation*.

[61] Garcia LA, Lyden SP (2009). Atherectomy for infrainguinal peripheral artery disease. *Journal of Endovascular Therapy*.

[62] Al Khoury G, Chaer R (2011). Evolution of atherectomy devices. *Journal of Cardiovascular Surgery*.

[63] Steed DL, Donohoe D, Webster MW, Lindsley L (1996). Effect of extensive debridement and treatment on the healing of diabetic foot ulcers. *Journal of the American College of Surgeons*.

[64] Sumpio BE, Armstrong DG, Lavery LA, Andros G (2010). The role of interdisciplinary team approach in the management of the diabetic foot a joint statement from the society for vascular surgery and the american podiatric medical association. *Journal of the American Podiatric Medical Association*.

[65] Granick M, Boykin J, Gamelli R, Schultz G, Tenenhaus M (2006). Toward a common language: surgical wound bed preparation and debridement. *Wound Repair and Regeneration*.

[66] Klein MB, Hunter S, Heimbach DM (2005). The Versajet water dissector: a new tool for tangential excision. *Journal of Burn Care and Rehabilitation*.

[67] Bergman-Evans B, Cuddigan J, Bergstrom N (1994). Clinical practice guidelines: prediction and prevention of pressure ulcers. *Journal of Gerontological Nursing*.

[68] Singer AJ, Clark RAF (1999). Cutaneous wound healing. *New England Journal of Medicine*.

[69] Blume P, Driver VR, Tallis AJ (2011). Formulated collagen gel accelerates healing rate immediately after application in patients with diabetic neuropathic foot ulcers. *Wound Repair and Regeneration*.

[70] Bello YM, Phillips TJ (2000). Recent advances in wound healing. *Journal of the American Medical Association*.

[71] Sumpio B, Driver VR, Gibbons GW (2009). A multidisciplinary approach to limb preservation: the role of V.A.C. therapy. *Wounds*.

[72] Blume P, Salonga C, Garbalosa J (2007). Predictors for the healing of transmetatarsal amputations: retrospective study of 91 amputations. *Vascular*.

[73] Bus SA, Valk GD, van Deursen RW (2008). The effectiveness of footwear and offloading interventions to prevent and heal foot ulcers and reduce plantar pressure in diabetes: a systematic review. *Diabetes/Metabolism Research and Reviews*.

[74] Bus SA, Valk GD, van Deursen RW (2008). Specific guidelines on footwear and offloading. *Diabetes/Metabolism Research and Reviews*.

[75] Armstrong DG, Lavery LA, Stern S, Harkless LB (1996). Is prophylactic diabetic foot surgery dangerous?. *Journal of Foot and Ankle Surgery*.

[76] Catanzariti AR, Blitch EL, Karlock LG (1995). Elective foot and ankle surgery in the diabetic patient. *Journal of Foot and Ankle Surgery*.

[77] Barrow J, Hughes J, Clarke P, Klenerman L (1992). A study of the effect of wear on the pressure-relieving properties of foot orthosis. *The Foot*.

[78] Nawoczenski DA, Birke JA, Coleman WC (1988). Effect of rocker sole design on plantar forefoot pressures. *Journal of the American Podiatric Medical Association*.

[79] Tang PC, Ravji K, Key JJ, Mahler DB, Blume PA, Sumpio B (2008). Let them walk! Current prosthesis options for leg and foot amputees. *Journal of the American College of Surgeons*.

[80] Orgill DP, Manders EK, Sumpio BE (2009). The mechanisms of action of vacuum assisted closure: more to learn. *Surgery*.

[81] Blume PA, Walters J, Payne W, Ayala J, Lantis J (2008). Comparison of negative pressure wound therapy usingVacuum-assisted closure with advanced moist wound therapy in the treatment of diabetic foot ulcers. *Diabetes Care*.

[82] Vig S, Dowsett C, Berg L (2011). Evidence-based recommendations for the use of negative pressure wound therapy in chronic wounds: steps towards an international consensus. *Journal of Tissue Viability*.

[83] Blume PA, Key JJ, Thakor P, Thakor S, Sumpio B (2010). Retrospective evaluation of clinical outcomes in subjects with split-thickness skin graft: comparing V.A.C. therapy and conventional therapy in foot and ankle reconstructive surgeries. *International Wound Journal*.

[84] Sumpio B, Thakor P, Mahler D, Blume P (2011). Negative pressure wound therapy as postoperative dressing in below knee amputation stump closure of patients with chronic venous insufficiency. *Wounds*.

[85] Sheehan P, Jones P, Caselli A, Giurini JM, Veves A (2003). Percent change in wound area of diabetic foot ulcers over a 4-week period is a robust predictor of complete healing in a 12-week prospective trial. *Diabetes Care*.

[86] Margolis DJ, Bartus C, Hoffstad O, Malay S, Berlin JA (2005). Effectiveness of recombinant human platelet-derived growth factor for the treatment of diabetic neuropathic foot ulcers. *Wound Repair and Regeneration*.

[87] Steed DL, Attinger C, Colaizzi T (2006). Guidelines for the treatment of diabetic ulcers. *Wound Repair and Regeneration*.

[88] Veves A, Falanga V, Armstrong DG, Sabolinski ML (2001). Graftskin, a human skin equivalent, is effective in the management of noninfected neuropathic diabetic foot ulcers: a prospective randomized multicenter clinical trial. *Diabetes Care*.

[89] Boulton AJ, Kirsner RS, Vileikyte L (2004). Clinical practice. Neuropathic diabetic foot ulcers. *The New England Journal of Medicine*.

[90] Tan T, Shaw EJ, Siddiqui F (2011). Inpatient management of diabetic foot problems: summary of NICE guidance. *British Medical Journal*.

[91] McMurray SD, McDougall K (2003). Improving diabetes foot care in the dialysis facility. *Nephrology News & Issues*.

[92] Boulton AJ (2000). The diabetic foot: a global view. *Diabetes/Metabolism Research and Reviews*.

[93] Stockl K, Vanderplas A, Tafesse E, Chang E (2004). Costs of lower-extremity ulcers among patients with diabetes. *Diabetes Care*.

[94] Lavery LA, Armstrong DG, Wunderlich RP, Mohler MJ, Wendel CS, Lipsky BA (2006). Risk factors for foot infections in individuals with diabetes. *Diabetes Care*.

[95] Ellison DA, Hayes L, Lane C, Tracey A, McCollum CN (2002). Evaluating the cost and efficacy of leg ulcer care provided in two large UK health authorities. *Journal of Wound Care*.

[96] Lloyd A, Sawyer W, Hopkinson P (2001). Impact of long-term complications on quality of life in patients with type 2 diabetes not using insulin. *Value in Health*.

[97] Ragnarson Tennvall G, Apelqvist J (2000). Health-related quality of life in patients with diabetes mellitus and foot ulcers. *Journal of Diabetes and Its Complications*.

[98] Goodridge D, Trepman E, Embil JM (2005). Health-related quality of life in diabetic patients with foot ulcers: literature review. *Journal of Wound, Ostomy, and Continence Nursing*.

[99] Ashford R, McGee P, Kinmond K (2000). Perception of quality of life by patients with diabetic foot ulcers. *Diabetic Foot*.

[100] Browse NL (1986). The etiology of venous ulceration. *World Journal of Surgery*.

[101] Goldman M, Fronek A (1992). Consensus paper on venous leg ulcer. *Journal of Dermatologic Surgery & Oncology*.

[102] Phillips T, Stanton B, Provan A, Lew R (1994). A study of the impact of leg ulcers on quality of life: financial, social, and psychologic implications. *Journal of the American Academy of Dermatology*.

[103] Sumpio B, Allie D, Horvath K (2008). Role of negative pressure wound therapy in treating peripheral vascular graft infections. *Vascular*.

